# The genetic diversity of chicken breeds from Jiangxi, assessed with BCDO2 and the complete mitochondrial DNA D-loop region

**DOI:** 10.1371/journal.pone.0173192

**Published:** 2017-03-03

**Authors:** Yu-shi Gao, Xiao-xu Jia, Xiu-jun Tang, Yan-feng Fan, Jun-xian Lu, Sheng-hai Huang, Meng-jun Tang

**Affiliations:** Jiangsu institute of Poultry Science, Yangzhou, China; National Cheng Kung University, TAIWAN

## Abstract

The Jiangxi Province of China has numerous native domestic chicken breeds, including some black skin breeds. The genetic diversity of Jiangxi native chickens is largely unknown, and specifically, the genetic contribution of the grey junglefowl to black skin chickens is not well understood. To address these questions, the complete D-loop region of the mitochondrial DNA (mtDNA) and beta-carotene dioxygenase 2(BCDO2)gene was sequenced in a total of 209 chickens representing seven Jiangxi native breeds. Thirty-one polymorphic sites were identified across the complete mtDNA D-loop region sequence. Twenty-three haplotypes were observed in the seven breeds, which belonged to four distinct mitochondrial clades (A, B, C and E). Clade A and B were dominant in the chickens with a frequency of approximately 67.9%. There were five SNPs that defined two haplotypes, W and Y in BCDO2. Four breeds had one haplotype and three breeds had two. We conclude that Jiangxi native chicken breeds have relatively low genetic diversity and likely share four common maternal lineages from two different maternal ancestors of junglefowl. Furthermore, some Jiangxi chicken populations may have been mixed with chickens with exotic lineage. Further research should be established to protect these domestic chicken resources.

## Introduction

Given its diversified geographical conditions and long history of animal husbandry, China is particularly rich in chicken genetic resources. There are a total of 107 local chicken breeds recorded in the National genetic resources[1,2]. Most of these breeds have not been selected intensively and have lower production performance than the commercial breeds. Although some native chicken breeds are not as economically valuable as commercial breeds, they remain an important genetic resource because they have been artificially selected over their long breeding history using criteria that differ greatly from those used for commercial breeds[3].

Today, commercial lines and industrialized livestock production systems have spread worldwide. Increased global use of highly productive commercial chicken breeds has reduced genetic diversity in some native breeds. During thousands of years of natural selection, the native chicken breeds have mostly adapted to the local environment. Genetic diversity within a species similarly increases the probability of survival in a range of environments[4]. Reducing the genetic diversity of a species means losing not only genetic ‘wealth’, but also reducing the possibility of the species to adapt to harsh environmental conditions and disease outbreaks[5]. Therefore, conservation of genetic diversity of domestic animals is important for breeding demands in the future[6].

Jiangxi is a southern province of China, extending from the banks of the Yangtze River in the north into the hillier areas in the south. Jiangxi has numerous native domestic chicken breeds with a wide range of different characteristics. These breeds have better meat quality, better immunity to diseases, better adaptability to extensive management, and are natural gene pools, thus making them good candidates for crossbreed predominance and high performance[7].The number of Jiangxi native chickens is decreasing owing to introgression of commercial breeds. Measures should be established to protect these domestic chicken resources.

Most studies that have assessed the phylogenetic relationships, genetic diversity and maternal origin of the domestic chicken rely on a partial sequence of the D-loop[8,9]. Although the whole mtDNA genome of a few Chinese chicken breeds has been sequenced [10], there has been no comprehensive analysis to evaluate the complete sequence of mtDNA D-loop genetic variability and relationships with large numbers of native Chinese chickens.

A study concerning identification of the yellow skin gene yielded new insights into the origins and history of chicken domestication. Yellow skin breeds were found to cluster with the grey junglefowl (*Gallus sonneratii*) in a phylogenetic analysis based upon the BCDO2 sequence. In contrast, white skin breeds were clustered with the red junglefowl (*Gallus gallus*)[11].SNPs (single nucleotide polymorphism) are also widely distributed in BCDO2in chicken. These nuclear markers may provide valuable additional information about the demographic history and population structure in chickens. Chickens in western countries have white or yellow skin, while some indigenous Chinese domestic chickens have black skin. We expected to observe a diversity of BCDO2among the black skin populations.

We generated the complete mtDNA D-loop sequences and BCDO2 for seven domestic chicken breeds from the Jiangxi province of China. This study has advanced the information on their genetic diversity and variation at the nuclear and mitochondrial level.

## Materials and methods

### Ethics statement

The chickens were obtained from conservation farms or conservation zones. All procedures were approved by the Animal Care and Use Committee of Jiangsu Institute of Poultry Science(permit number: SYXK 2016–0020).

### Animals, sample collection and DNA extraction

The chickens were from seven local breeds distributed across the Jiangxi Province (Anyi Gray chicken(AY), n = 30; Baier Yellow chicken(BE), n = 30; Chongren Partridge chicken(CP), n = 30; Dongxiang Blue-eggshell chicken(DX), n = 30; Jinhu Black-bone chicken(JH), n = 29; Silkie chicken(SL), n = 30; Xianju chicken(XJ), n = 30).

**Fig 1:**
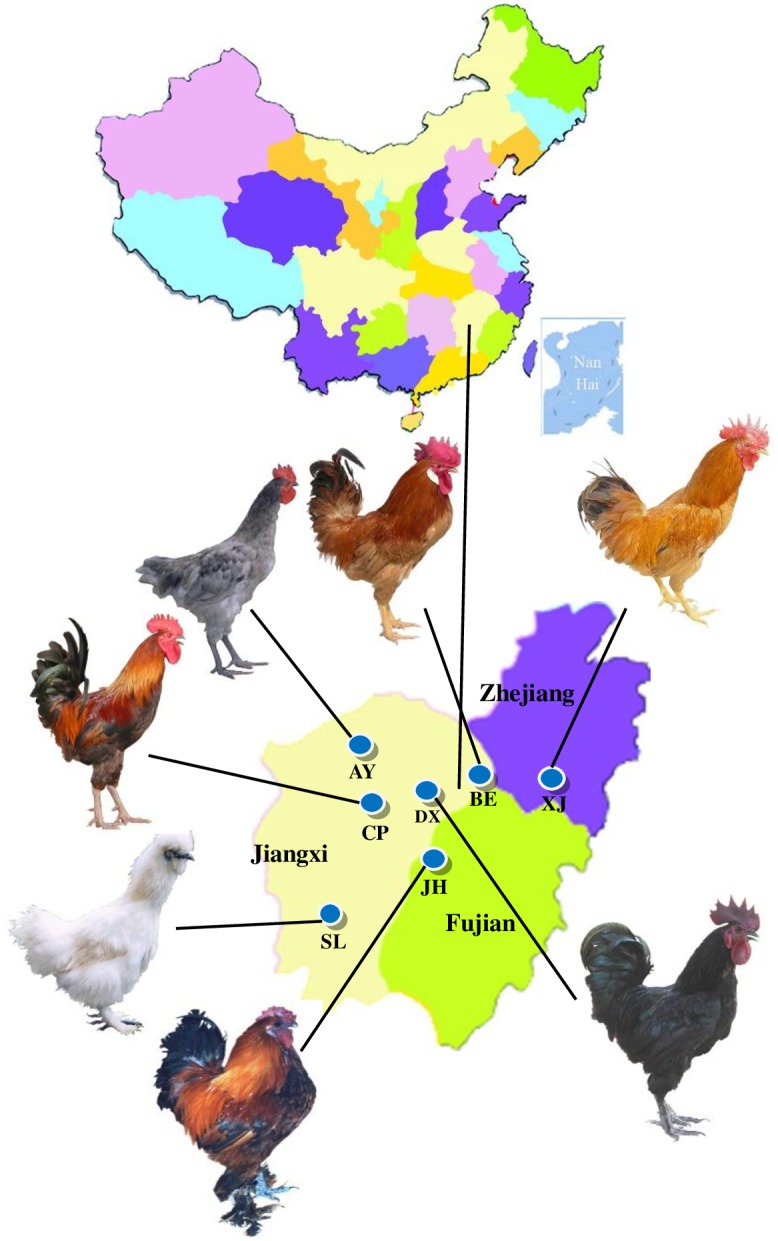
Geographic distribution of and males of the seven breeds used in the current study.

In total, 209 blood samples were collected from the seven breeds, as described below in Table 1 and Fig 1. Genomic DNA was extracted from these blood samples using the standard phenol/chloroform method[12].

**Table 1:** Conservation status and characteristics of the seven domestic chicken breeds.

Breed (abbrev)	Population size(sample size)	Longitude and latitude	Economic type	Main skin color	Main shank color	Main beak color	Main feather color
**Anyi Gray (AY)**	600000(30)	28°36′~29°01′N;115°27′~115°45′E	E&M	Gray	Gray	Gray or black	Gray
**Baier Yellow (BE)**	20000000(30)	28°3′~28°37′N;118°1′~118°29′E	E	Yellow	Yellow	Yellow	Yellow
**Chongren Partridge (CP)**	52000000(30)	27°25′~27°56′N;115°49′~116°17′E	E&M	White	Black	Black	hen spotty, cock red with black
**Dongxiang Blue-eggshell(DX)**	200000 (30)	28°14′N;116°36′E	E&M	Black	Black	Black	Black
**Silkies(SL)**	16500000(30)	26°27′N;114°57′E	Med&F&M	Black	Black	Black	white
**Jinhu Black-bone(JH)**	160000(29)	26°34′~27°07′N;116°34′~117°24′E	Med&M	Black	Black	Black	hen spotty, cock red with black
**Xianju(XJ)**	1484000(30)	28°51′N;120°44′E	E	Yellow	Yellow	Yellow	Yellow

M = meat, E = egg, Med = medical, F = fancy.

### PCR amplification and DNA sequencing

The complete chicken mtDNA D-loop region was amplified using the primers from Jia. et al.[13].Based on the *Gallus gallus* BCDO2 sequence (GenBank accession no. EU334166), primer pairs were designed to correspond to the SNP (chr24:6,273,428A<G) in complete linkage disequilibrium with yellow skin. These were P1(sense) 5'-AGGAAGGGAAGGATTAGC-3' andP2 (antisense)5'-TTATGTGCTCGCAGAATG-3', synthesized and purified by Invitrogen Biotechnology Co., Ltd (Shanghai, China).PCR was performed in a 50 μL reaction volume containing 25 μL 2×Taq Master Mix (Body Science, Nanjing, China), 50–100 ng template DNA, 10 pM of each primer, and 21 μL RNase-Free water. The PCR amplification conditions were as follows: 95°C for 30 s, followed by 35 cycles of 95°C for 30 s, 53°C for 60 s (BCDO2 annealing at 55°C) and 72°Cfor 60 s, followed by a final extension for 10 min at 72°C.The PCR products were purified from agarose gel after electrophoresis and sequenced using the ABI 3130 DNA sequencer by primer walking (Applied Biosystems, Foster City, CA, USA).

### Data analysis

All reads(mtDNA and BCDO2)obtained were assembled and aligned using the DNAstar package (DNASTAR, Inc., Madison) against the reference sequence (NC007235 or EU334166).Sequences with phred quality value of at least 20wereconsidered for further analysis.

Neighbor-Joining (NJ) method was used to analyze the evolutionary relationships using MEGA software (version 5.0)[14]. The number of haplotypes, average number of nucleotide differences (k), haplotype diversity (Hd) and nucleotide diversity (p)were determined using the DnaSP program (version 5.10) [15]. A median-joining network was constructed to determine the evolutionary relationships of haplotypes using the default settings provided in NETWORK (version 5.0) [16].

## Results

### Genetic variation of the complete mtDNA D-loop

The D-loop regions of the native breeds were 1231–1232 bp in length. The 1231 bp haplotype had 119 sequences with base C deficiency at 859 bp. The 1232 bp haplotype had 90 sequences. All mtDNA D-loop sequences obtained were deposited into GenBank with accession numbers KY307966–KY308174.

**Fig 2:**
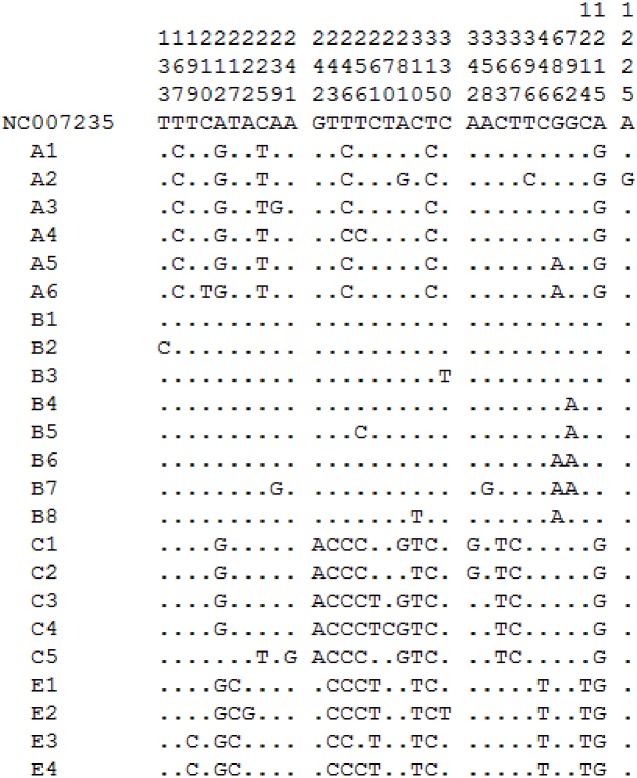
Variable sites in mtDNA D-loop of 23 haplotypes derived from 209chickens. Dots (.) denote sequences identical to the reference sequence (NC007235).

In 209 chickens, the average nucleotide composition was 33.5% T, 26.6% A, 26.5% C, and 13.3% G, and the content of AT (60.1%) was significantly greater than that of GC (39.9%), showing some base bias. There were 31 polymorphic sites with three singleton polymorphic sites and 28 parsimony-informative polymorphic sites, as shown in Fig 2.

We did not find any nucleotide mutations from site 1 to 132 bp, while 133 to 446 bp was a relatively variable region with 26 mutations mapping to this region. 447 to 1232 bp was a relatively conserved region having five variable sites (686, 792, 1214, 1215, and 1225 bp).

The 686(G-A) variable site was found only in Clades A and B, while the 792(G-A) variable site was found only in Clade B, almost simultaneously with 686(G-A). All animals of the Clade E had a C-T base transition at 1214 bp. Clades A, C and E showed an A-G transition. A single-base deletion was found at 859 bp in all four Clades. The deletion regularities were not found.

### Genetic diversity and distance among seven breeds

The calculated number of haplotype diversity and polymorphic sites from the seven breeds are shown in Table 2. The overall nucleotide diversity of the seven breeds was 0.00591 ± 0.00111, ranging from 0.00221 ± 0.00075 in SL to 0.00615 ±0.00174 in JH. The overall haplotype diversity was 0.893± 0.011. The greatest haplotype diversity was in AY (0.837 ± 0.027) whereas the lowest was in SL (0.467 ± 0.087). The overall nucleotide differences was 7.051, ranging from 2.720 in SL to 7.540 in AY.

**Table 2:** mtDNA genetic diversity index of the seven domestic chicken breeds.

Population	Sample size	Number of haplotypes	Haplogroup (number of individuals observed)	Haplotype diversity (SD)	Nucleotide diversity (SD)	Mean number of nucleotide differences
**AY**	30	6(A = 1;B = 2;C = 3)	A(3);B(12);C(15)	0.837(0.027)	0.00613 (0.00140)	7.540
**BE**	30	6(A = 1;B = 4;C = 1)	A(8);B(21);C(1)	0.680(0.066)	0.00424(0.00133)	5.218
**CP**	30	7(A = 1;B = 3;C = 1;E = 2)	A(2);B(23);C(1);E(4)	0.736(0.054)	0.00425(0.00178)	5.232
**DX**	30	4(A = 1;B = 1;C = 1;E = 1)	A(13);B(4);C(2);E(11)	0.678(0.049)	0.00457(0.00138)	5.444
**JH**	29	6(A = 2;B = 1;C = 1;E = 2)	A(3);B(11);C(1);E(14)	0.670 (0.057)	0.00615(0.00174)	7.571
**SL**	30	3(A = 2;B = 1)	A(23);B(7)	0.467(0.087)	0.00221(0.00075)	2.720
**XJ**	30	5(A = 1;B = 3;C = 1)	A(8);B(4);C(18)	0.582(0.079)	0.00492(0.00127)	6.053
**All**	209	23(A = 6;B = 8;C = 5;E = 4)	(A = 60;B = 82;C = 38;E = 29)	0.893(0.011)	0.00591(0.00111)	7.051

SL has been bred for entertainment purposes over many years. They have been selected for standard characteristics of a strawberry comb, phoenix head, green ears, beard, silky plumage, five toes, feathered-feet, black skin, black muscle, and black shanks. High selection pressure may be the reason why SL had the lowest nucleotide diversity values. AY is a newly found breed that was included in the National breed protection list in 2009. It has been exploited and utilized less, so it has great genetic diversity.

The genetic distances within and between the seven chicken breeds are presented in Table 3. Within the seven chicken breeds, the genetic distance was 0.0020~0.0060. AY chickens had the highest within-breed genetic distance, while SL chickens had the lowest. Between the breeds, the genetic distance values ranged from 0.0042 to 0.0082. The genetic distance between the breeds was greatest for XJ and BE chickens, while it was lowest for DX and SL chickens.

**Table 3:** Kimura 2-parameter genetic distance between each pair among the seven chicken breeds.

**Breed**	**With breeds**	**AY**	**BE**	**CP**	**DX**	**JH**	**SL**	**XJ**
**AY**	0.0060							
**BE**	0.0040	0.0072						
**CP**	0.0038	0.0061	0.0050					
**DX**	0.0046	0.0069	0.0069	0.0058				
**JH**	0.0056	0.0072	0.0065	0.0055	0.0059			
**SL**	0.0020	0.0061	0.0050	0.0049	0.0042	0.0060		
**XJ**	0.0049	0.0059	0.0082	0.0071	0.0066	0.0074	0.0062	

### Phylogenetic relationships

**Fig 3:**
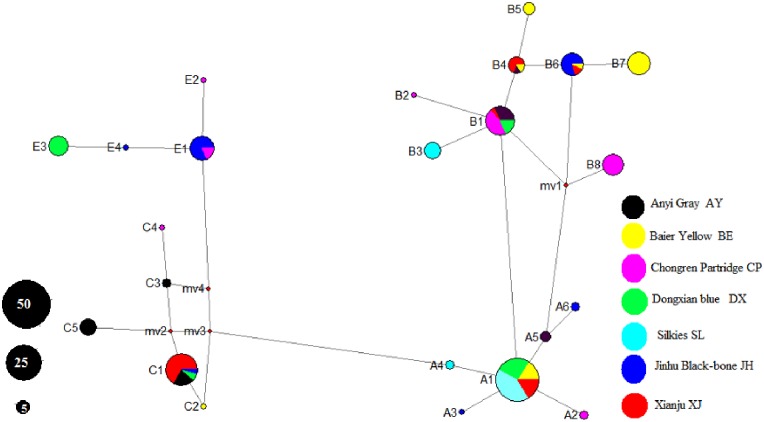
Median network profile of the mtDNA D-loop haplotypes observed in the present study. The areas of the circles represent haplotype frequencies.

The median-joining network was constructed using the 23 haplotypes of this study(Fig 3).Haplotypes found in the present study were compared to previous clades defined by Liu et al. [9]. Four clades were observed, namely, A, B, C and E. Six haplotypes were observed for Clade A, eight for Clade B, and four each for Clades C and E. Clade B is relative larger than the other clades and accounted for 39.2% of all samples, which contained 82 individuals. This was followed by Clade A (28.7%, 60 individuals), C (18.2%, 38 individuals) and E (13.9%, 29 individuals).

No single haplotype was shared by all seven populations as shown in Table 4. In each of the four clades (A, B, C, and E), there was a dominant haplotype. A major haplotype (A1) occurred at a frequency of 23.9% (50/209), distributed in four local populations (BE8, DX13, SL21, XJ8). Likewise, the second major haplotype (C1) occurred at a frequency of 12.9% (27/209) and was observed in four chicken populations (XJ18, AY6, DX2, and JH1). The third major haplotype (B1) was distributed in four chicken populations (XJ1, AY7, CP10, DX4) at a frequency of 10.5% (22/209). E1 was distributed in two chicken breeds, each with 16 individuals (JH13, CP3).

**Table 4:** Numbers of individuals with each haplotype.

Haplotype	Breed and number of individuals^a^	Total number of chickens with the haplotype
**A1**	BE8,DX13,SL21,XJ8	50
**A2**	CP2	2
**A3**	JH1	1
**A4**	SL2	2
**A5**	AY3	3
**A6**	JH2	2
**B1**	XJ1, AY7, CP10, DX4	22
**B2**	CP1	1
**B3**	SL7	7
**B4**	XJ5, AY1, BE1	7
**B5**	BE4	4
**B6**	JH11, XJ2, BE1	14
**B7**	BE15	15
**B8**	CP12	12
**C1**	XJ18, AY6, DX2, JH1	27
**C2**	BE1	1
**C3**	AY2	2
**C4**	CP1	1
**C5**	AY7	7
**E1**	JH13, CP3	16
**E2**	CP1	1
**E3**	DX11	11
**E4**	JH1	1
**all**		209

^a.^ Names of breeds corresponding to these abbreviations are given in Table 1; the number in the column after the abbreviation indicates how many chickens carried the indicated haplotype.

### Genetic variation and haplotype sharing of BCDO2

There were five SNPs that defined two haplotypes W and Y (Table 5) SL and CP both had haplotype W, although SL has black skin while CP had white skin, Common characteristics of the two breeds are black shanks and beaks. BE and XJ had haplotype Y. They both have yellow skin, shanks and beaks. AY, DX and JH have two haplotypes. The Y haplotype was found in high proportions among the AY and DX individuals, whereas JH had a high proportion of the W haplotype. DX and JH have black skin and shanks; AY have gray skin and shanks.

**Table 5:** BCDO2 genetic diversity index of the seven domestic chicken breeds.

	SNP position					Number of haplotypes (percentage) per breed						
Haplotype	6273	6273	6273	6273	6273	AY	BE	CP	DX	JH	SL	XJ
	91	129	229	240	428							
**W**	G	A	C	C	A	10(33.3%)		30(100%)	11(36.7%)	22(73.3%)	30(100%)	
**Y**	A	G	T	T	G	20(66.7%)	30(100%)		19(63.3%)	7(16.7%)		30(100%)

## Discussion

### Structural features of the complete mtDNA D-loop

The complete sequences of mtDNA D-loop from 209 individuals in seven chicken breeds were 1231–1232 bp long, which was longer than duck (1050 bp) and goose (1178 bp), but shorter than pigeon (1656 bp)(www.ncbi.nlm.nih.gov) indicating a rapid evolutionary rate.

Most previous studies found that variable sites in the D-loop were between 133–446 bp long, or less. The mutation rate in this region is higher than in the others.

We detected five variable sites (686, 792, 1214, 1215, 1225 bp) between 447~1232 bp. The 1225 bp (A-G)site has not been detected previously, and was therefore re-sequenced to confirm its presence. The complete sequence of mtDNA D-loop provides more details than the partial sequence. Recently, the complete mtDNA genome was used to reconstruct the genetic development of animal domestication in species including pigs[17],sheep[18],dogs[19] and horses[20].However, the investigation of genetic diversity and evolution of animals requires large numbers of animals for the results to be representative. Analyzing large numbers of complete mitogenomes is expensive. Therefore, the complete sequence of the D-loop may provide useful genetic markers to study population genetics and trace the origins of domestic animals.

Along with the progress of sequencing techniques, there has been an increase in the accumulation of mitochondrial genome sequences from domestic animals. Mitochondrial genomes of domestic animals need to be scrutinized [21].Many sequences may have sequencing errors, such as extensive indels and ambiguous sites[11]. We recommend considering sending the sequence directly to GenBank, with correct annotation before submission[22].

### Genetic diversity and origin of seven domestic chicken breeds

The mtDNA D-loop is widely used in the study of genetic diversity of chickens. Among the seven domestic breeds studied, haplotype diversity ranged from 0.467 to 0.837, lower than that of breeds in Guangxi (0.515 to 0.908)[23],Yunnan (0.545 to 0.922)[24] and Laotian (0.815 to 0.880) [25], and similar to that of Korean(0.590 to 0.820) [26] breeds. The genetic diversity of the Jiangxi native chicken breeds is relatively low. When compared with commercial breeds, most native chickens have low productivity. Reducing number of individuals of these breeds is one of the causes of reduction in diversity[27].Artificial selection practices can also reduce diversity[28].In our study, the SL and XJ breeds have lower diversity.SL chickens have been selected for ten standard characteristics for many years. In China, most native chicken breeds are dual purpose; however, XJ is a breed, which has been bred for egg laying. Artificial selection pressure may be the reason why SL and XJ breeds have lower genetic diversity.

When an egg is fertilized, cells of the resulting embryo contain the cytoplasm and mtDNA of the egg. Thus, mtDNA has a direct lineage to the ancestral mother. mtDNA has been used to assess regions of domestication, identify their geographic origins and the number of maternal lineages[29].

The mtDNA D-loop phylogeny extends our understanding of the matrilineal history of poultry. When compared with other poultry, sequence variation in chicken mtDNA, particularly in the polymorphic region of D-loop, is greater. Previous work indicated that the Chinese domestic ducks and geese have two maternal origins. Ducks were mainly derived from the mallard (*Anas platyrhynchos*), while a few derived from the spot-billed duck (*Anas zonorhyncha*)[30]. Geese breeds mainly originated from the swan goose (*Anser cygnoides*) and a few originated from the greylag goose (*Anser anser*)[7]. Chinese domestic chickens have at least nine maternal origins, seven of which consist of both red junglefowl and domestic chicken subspecies[9]. Because waterfowl lives in or near water whereas the ancestors of domestic chickens lived on land, the ancestors of chickens were better adapted to their environment, thereby allowing them to have more offspring.

The chicken breeds in the present study were found in four clades of mtDNA (A, B, C and E). We did not detect the five Clades D, F, G H and I described by Liu et al. [9]. This result is consistent with Guangxi chicken breeds [23] and Tanwan chicken breeds[6]. Clade D could be related to the distribution of game birds. F and G clades were only found in Yunnan, China. Clade H was mainly present in red junglefowl and Clade I was found only in Vietnam[9].The absence of Clades D, F, G, H and I in Jiangxi chickens may be expected owing to its distance from the center of the domestication event for chicken, which was Southeast Asia. In these regions, wild junglefowl and domestic chickens may have experienced substantial genetic admixture and gene flow following domestication[10]. Furthermore some domestic chickens might have become feral with their descendants living as wild fowl[29].

Based on the current understanding of the regions where chicken domestication originated, mtDNA results suggest the AY and XJ breeds possess a dominant maternal lineage (C) and two minor lineages (A and B). BE possesses a dominant maternal lineage (B) and two minor lineages (A and C). CP possesses a dominant maternal lineage (B) and three minor lineages (A, C and E). DX possesses two dominant maternal lineages (A and E) and two minor lineages (B and C). JH possesses two dominant maternal lineages (B and E) and two minor lineages (A and C). Only the SL breed has two maternal lineages of which A is dominant.

Clade A and B were dominant in the 209 chickens representing the seven native breeds with a frequency of approximately 67.9%. Haplogroups A and B are distributed worldwide, except Africa, and originated from Yunnan and/or its surrounding area[9]. Clade C contained six breeds; the only breed missing from this clade was SL. In the AY and XJ breeds, individuals of Clade C were greater than or equal to half of the populations. In the other four breeds, each breed had only one or two individuals. Haplogroup C is mainly distributed in East Asia, Southeast Asia and South Asia[9]. It is difficult to identify breed-specific mtDNA marker, presumably because chicken is easily transported and has frequently been used to investigate migration, admixture and domestication[10].

The fewest individuals and haplotypes were in Clade E, which was shared by three breeds, i.e. CP, DX and JH. This haplogroup is distributed ubiquitously in modern European chickens (and Western commercial breeds).The presence of Clade E suggests that three breeds may have been admixed with highly productive European breeds. Highly productive commercial breeds were introduced into China in recent decades[1] and crossbred with native breeds to improve production of the native breeds. Introgression of commercial breeds into indigenous populations may have led to a loss of biodiversity, thereby limiting the flexibility of future breeding programs. The domestic chicken resources of Jiangxi, especially for the CP, DX and JH breeds, should be protected.

### Diversity of BCDO2 among seven domestic chicken breeds

In China, chickens with yellow skin usually have yellow shanks, beaks and feathers; in the present study, the BE and XJ were of this type. Chickens with white skin mostly have black shanks, beaks and feathers, although the hen’s feather color is mostly partridge; in the present study, the CP was of this type. This difference may be due to tissue-specific regulatory mutations that inhibit expression of the BCDO2 enzyme, thereby allowing accumulation of yellow carotenoids in the skin, shanks, beak and feathers. Therefore, BCDO2 is not the candidate gene for only skin color, but may also affect shanks, beak and feather color. People in southern China prefer three-yellow chicken (i.e. yellow shanks, feathers and beak). Because Chinese people are fond of buying live birds, packaging traits (feather, shank and beak color) are more important than skin color. Yellow skin may be a by-product, which arose during artificial selection for yellow packaging traits. Mutations in BCDO2 also influence carotenoid accumulation in other livestock. In sheep, a yellow fat phenotype may be caused by a nonsense mutation in the BCDO2 gene, which results in high levels of carotenoids being deposited in fat[31]. In cows, a mutation in the BCDO2 gene results in increased β-carotene concentrations in both milk and serum[32].

Based on the variation of the BCDO2 gene in domestic chickens and closely related wild species, Eriksson et al. [11] contradicted the suggestion that the red junglefowl is the sole wild ancestor of the domestic chicken. The yellow skin allele may originate from a species of junglefowl other than the red junglefowl, most likely, the grey junglefowl. In China, half the chicken breeds are like the CP breed, having white skin, mostly with black shanks and beaks. These are typical characteristics of the red junglefowl, indicating that these breeds may originate from this species. The yellow skin chickens may have originated from grey junglefowl, or their sole wild ancestor might now be extinct. Therefore, the red junglefowl is widely believed to be the primary ancestor of the present domestic chicken, although genetic contributions from the other junglefowl cannot be excluded[33].Nuclear markers (such as BCDO2, MHC and TSHR) may provide valuable additional information with respect to the domestication of chickens[34].

For most countries in the world, the majority of chickens have been bred for two purposes, egg laying and meat production. However, in some areas of China, black skin chickens were used in traditional Chinese medicine. Because of this different use, China has bred various varieties of black skin chicken. There are 17 black skin chicken breeds included in the National breed protection list. In the present study (see Table 5), four black skin breeds, and the SL breeds have the W haplotype. The AY, DX and JH breeds have W and Y haplotypes. The characteristics they shared were black skin, bone, muscle and shanks. SL is different in that it has white feathers whereas the other breeds are colored (AY is grey, DX is black, JH is partridge). Possibly, BCDO2is only one major gene functioning in skin color, and several genes function in black skin chicken. The black skin chickens may have at least two different maternal ancestors of junglefowl.
